# Radiation therapy for cancer is potentially associated with reduced growth of concomitant abdominal aortic aneurysm

**DOI:** 10.1007/s00066-023-02135-0

**Published:** 2023-09-07

**Authors:** Aaron Becker von Rose, Kathrin Kobus, Bianca Bohmann, Moritz Lindquist-Lilljequist, Wolf Eilenberg, Marvin Kapalla, Florian Bassermann, Christian Reeps, Hans-Henning Eckstein, Christoph Neumayer, Christine Brostjan, Joy Roy, Korbinian von Heckel, Rebecka Hultgren, Benedikt J. Schwaiger, Stephanie E. Combs, Albert Busch, Kilian Schiller

**Affiliations:** 1 III. Medical Department for Hematology and Oncology, University Hospital rechts der Isar, Technical University Munich, Munich, Germany; 2 Department for Vascular and Endovascular Surgery, University Hospital rechts der Isar, Technical University Munich, Munich, Germany; 3 Stockholm Aneurysm Research Group (STAR), Department of Vascular Surgery, Karolinska Institutet and Karolinska University Hospital, Stockholm, Sweden; 4 Division of Vascular Surgery, Department of General Surgery, Medical University of Vienna and University Hospital Vienna, Vienna, Austria; 5 Division of Vascular and Endovascular Surgery, Department for Visceral‑, Thoracic and Vascular Surgery, Medical Faculty Carl Gustav Carus and University Hospital, Technische Universität Dresden, Dresden, Germany; 6 Department of Biology II, University of Munich (LMU), Munich, Germany; 7 Department of Diagnostic and Interventional Neuroradiology, School of Medicine, University Hospital rechts der Isar, Technical University Munich, Munich, Germany; 8 Department of Radiation Oncology, University Hospital rechts der Isar, Technical University Munich, Munich, Germany

**Keywords:** Radiotherapy, Aneurysm growth, Cardiovascular disease, Neoplasms, Radiation exposure

## Abstract

**Purpose:**

Co-prevalence of abdominal aortic aneurysm (AAA) and cancer poses a unique challenge in medical care since both diseases and their respective therapies might interact. Recently, reduced AAA growth rates were observed in cancer patients that received radiation therapy (RT). The purpose of this study was to perform a fine-grained analysis of the effects of RT on AAA growth with respect to direct (infield) and out-of-field (outfield) radiation exposure, and radiation dose-dependency.

**Methods:**

A retrospective single-center analysis identified patients with AAA, cancer, and RT. Clinical data, radiation plans, and aneurysm diameters were analyzed. The total dose of radiation to each aneurysm was computed. AAA growth under infield and outfield exposure was compared to patients with AAA and cancer that did not receive RT (no-RT control) and to an external noncancer AAA reference cohort.

**Results:**

Between 2003 and 2020, a total of 38 AAA patients who had received well-documented RT for their malignancy were identified. AAA growth was considerably reduced for infield patients (*n* = 18) compared to outfield patients (*n* = 20), albeit not significantly (0.8 ± 1.0 vs. 1.3 ± 1.6 mm/year, *p* = 0.28). Overall, annual AAA growth in RT patients was lower compared to no-RT control patients (1.1 ± 1.5 vs. 1.8 ± 2.2 mm/year, *p* = 0.06) and significantly reduced compared to the reference cohort (1.1 ± 1.5 vs. 2.7 ± 2.1 mm/year, *p* < 0.001). The pattern of AAA growth reduction due to RT was corroborated in linear regression analyses correcting for initial AAA diameter. A further investigation with respect to dose-dependency of radiation effects on AAA growth, however, revealed no apparent association.

**Conclusion:**

In this study, both infield and outfield radiation exposure were associated with reduced AAA growth. This finding warrants further investigation, both in a larger scale clinical cohort and on a molecular level.

**Supplementary Information:**

The online version of this article (10.1007/s00066-023-02135-0) contains supplementary material, which is available to authorized users.

## Introduction

With an increase in elderly patients due to rising life expectancy, the issue of multimorbidity and in particular the co-prevalence of cancer and cardiovascular diseases is becoming one of the major challenges in medical care [1, 2]. Depending on the type of cancer, up to almost 5% of patients are estimated to have concurrent disease affecting the circulatory system [3]. Abdominal aortic aneurysm (AAA) is the most common aneurysm of the aorta and is associated with an inherent risk of rupture, which despite surgical care proves fatal in ~75% of cases [4]. Approximately 5% of AAA patients have a concomitant malignant disease [5].

International guidelines recommend aneurysm treatment for cancer patients in line with noncancer patients, based on diameter threshold or growth rate irrespective of malignancy status and cancer therapy [6]. Specific types of cancer and cancer therapies, however, might be associated with a transformative effect on aneurysm growth and rupture risk.

While the effect of chemotherapies on AAA growth has been investigated in three large retrospective cohort studies, the effects of radiation on aneurysm growth are unknown [7–9]. However, radiation is part of more than 50% of modern anti-cancer regimens [10, 11]. Recently, our group detected a potentially beneficial effect of radiation therapy (RT) on aneurysm growth stability in a cohort of 217 patients [9]. Here, annual AAA growth rates were significantly reduced in cancer patients who received RT compared to cancer patients without RT and to a nonmalignancy reference cohort.

To shed further light on this unexpected finding, we re-analyzed this cohort regarding radiation plans and a dose-dependency of radiation effects on AAA growth stabilization. We hypothesized a possible local and/or systemic effect of RT in the aneurysm wall that warrants further elucidation regarding infield/outfield effects.

## Patients and methods

### Patient identification

Patients were retrospectively identified as described before [9]. Briefly, all cleared computed tomography (CT) examination reports in the institutional picture archiving and communication system (PACS) database from thorax, abdomen and/or pelvis examinations acquired between January 1, 2003 and March 31, 2020, were screened using a full-text query including the key words ‘aneurysm’ and ‘carcinoma’, ‘tumor’, ‘radiotherapy’, ‘radiation therapy’ and the different types of carcinomas (Suppl. Table 1). Patient data were pseudonymized for further analysis. Data are reported in accordance with Strengthening the Reporting of Observational Studies in Epidemiology (STROBE) criteria [12].

### Inclusion/exclusion criteria

Inclusion criteria were presence of a malignant tumor, in-house RT with detailed radiation plan on hand, and presence of an AAA (maximum transverse diameter > 30 mm) confirmed by CT angiography (CTA). Patients with an initial AAA diameter between 20–29 mm that surpassed 30 mm during the course of the study (*n* = 8) and patients with an initial diameter > 49 mm (*n* = 3) were analyzed but have been excluded from comparisons with other patient cohorts. AAA location was strictly abdominal, namely infra- (*n* = 36) and juxtarenal (*n* = 2). Patients with thoracic or thoracoabdominal aneurysms were excluded. In addition, for longitudinal aneurysm growth, a CT-based follow-up period of ≥ 6 months covering ≥ 2 individual CT(A) examinations was required.

Tumor diagnosis and RT had to overlap with AAA diagnosis, and patients were included if: (i) an ongoing RT started no more than 12 months before the baseline CT; (ii) RT was within the CT observation period; (iii) RT was initiated > 3 months before the last CTA.

Exclusion criteria were age < 18 years, incomplete clinical (no established tumor diagnosis) or imaging data, and diagnosis of aortic dissection or connective tissue disease.

As an in-house control for the effect of RT, AAA patients with concomitant malignancy that have not received RT (no-RT control) were identified using otherwise identical criteria.

An overview of the patient analysis and identification workflow is given in Suppl. Fig. 1. All patient cohorts are described in detail in Tables 1 and 2 and Suppl. Tables 2–4.

**Table 1:** Patient characteristics. Details on comorbidities and medication were not available for all patients. In these categories, percentages are given with respect to the number of patients for which this information was available

	RT^a^		Infield^a^		Outfield^a^		RT		Infield		Outfield		No-RT control		Noncancer reference	
	*n* = 38	%	*n* = 18	%	*n* = 20	%	*n* = 27	%	*n* = 13	%	*n* = 14	%	*n* = 62	%	*n* = 158	%
*Patient characteristics*																
Male	32	84.2	15	83.3	17	85.0	22	81.5	10	76.9	12	85.7	58	93.5	125	79.1
Age (years: mean ± SD)	71 ± 7.3		71 ± 8.5		70 ± 3.7		72 ± 6.6		74 ± 7.1		71 ± 5.8		69 ± 7.9		71 ± 8.2	
*Comorbidities*	26	68.4	13	72.2	13	65.0	19	70.4	10	76.9	9	64.3	44	71.0	158	100.0
Arterial hypertension	17	65.4	10	76.9	7	53.8	12	63.2	7	70.0	5	55.6	27	61.4	92	58.2
Coronary artery disease	11	42.3	5	38.5	6	46.2	7	36.8	3	30.0	4	44.4	19	43.2	47	29.7
Hyperlipidemia	9	34.6	4	30.8	5	38.5	7	36.8	3	30.0	4	44.4	15	34.1	72	45.6
Diabetes	8	30.8	3	23.1	5	38.5	6	31.6	3	30.0	3	33.3	8	18.2	36	24.7
Peripheral artery disease	1	3.8	1	7.7	0	0.0	1	5.3	1	10.0	0	0.0	9	20.5	21	13.3
Smoker (current/former)	11	42.3	6	46.2	5	38.5	7	36.8	5	50.0	2	22.2	26	59.1	134	84.8
*Medication*	20	52.6	9	50.0	11	55.0	14	51.9	7	53.8	7	50.0	15	24.2	158	100.0
Antiplatelets	13	65.0	7	77.8	6	54.5	9	64.3	5	71.4	4	57.1	7	46.7	107	67.7
Statins	8	40.0	2	22.2	6	54.5	4	28.6	1	14.3	3	42.9	7	46.7	82	51.9
Antihypertensives	8	40.0	2	22.2	6	54.5	5	35.7	1	14.3	4	57.1	7	46.7	83	52.5
Metformin	3	15.0	1	11.1	2	18.2	2	14.3	1	14.3	1	14.3	1	6.7	28	17.7
Insulin	1	5.0	1	11.1	0	0.0	1	7.1	1	14.3	0	0.0	2	13.3	5	3.2
*Observation time*																
Years (mean ± SD)	4.8 ± 3.5		5.5 ± 3.2		4.3 ± 3.8		4.0 ± 2.9		4.2 ± 2.5		3.8 ± 3.4		3.5 ± 3.1		3.2 ± 1.3	

*SD* standard deviation, *AAA* abdominal aortic aneurysm

^a^The radiation therapy (RT) cohorts marked with a superscript include patients with an initial AAA diameter between 20–29 and > 49 mm that were subsequently removed for comparison with the no-RT control and the reference

**Table 2:** Average and median annual AAA growth rates in the different patient cohorts. For all above cohorts, data stratified for initial aneurysm size classes is given in Suppl. Table 4

Patient cohort	RT^a^ (*n* = 38)	Infield^a^ (*n* = 18)	Outfield^a^ (*n* = 20)	RT (*n* = 27)	Infield (*n* = 13)	Outfield (*n* = 14)	No-RT control (*n* = 62)	Noncancer reference (*n* = 158)
Average annual AAA growth rate (mm/year)	1.06	0.79	1.31	1.09	0.73	1.42	1.80	2.72
SD	1.33	1.01	1.55	1.50	1.18	1.72	2.19	2.08
Median annual AAA growth rate (mm/year)	1.00	0.84	1.14	1.16	0.21	1.25	1.39	2.33
IQR	1.36	1.24	1.76	1.47	1.32	1.88	1.99	2.39

*SD* standard deviation, *AAA* abdominal aortic aneurysm, *IQR* interquartile range

^a^The radiation therapy (RT) cohorts marked with a superscript include patients with an initial AAA diameter between 20–29 and > 49 mm that were subsequently removed for comparison with the no-RT control and the reference

### Outcome parameters

The primary outcome was the average annual AAA growth rate in the study cohort with respect to RT (yes vs. no).

The secondary outcome was the AAA growth rate with respect to AAA location inside (infield) or outside (outfield) of the dose wash of the radiation field.

The tertiary outcome was the AAA growth rate with respect to the dosage of radiation.

In addition, the AAA growth rate based on initial diameter was studied in this AAA + cancer cohort in comparison to a noncancer AAA reference cohort.

### Stepwise analysis process

The included patients were individually assessed based on electronic patient files and medical records by two reviewers (ABR, KK) independently. For every CT(A) the aneurysm defined in the examination report was reviewed and a centerline-based leading edge transversal measurement of the maximum diameter was obtained by three independent reviewers (KK, BS, AB) using a three-dimensional (3D) multiplanar reconstruction (MPR), while two were completely blinded to the medical records (BS, AB). Consensus was reached upon discrepancy by joint reassessment [13]. All aneurysm growth data were calculated using the “first–last diameter/time” method as reviewed before [14, 15].

The patient baseline characteristics included sex, age, comorbidities (arterial hypertension, coronary artery disease, hyperlipidemia, diabetes, peripheral artery disease, connective tissue disease, and smoking status) and medication (antiplatelets, statins, antihypertensives, metformin, insulin). Specific malignancies were grouped together based on affected organ and prevailing malignancy based on the International Classification of Diseases (ICD; Suppl. Table 1). Tumor stages were summarized by the TNM staging system.

### Computation of aneurysmal radiation exposure

Every aneurysm was manually contoured by a specialist in radiation oncology (KS) using the available CT scans that had been created for RT treatment planning. Various metrics for aneurysmal radiation exposure were computed using Aria planning software (Varian Medical Systems Inc., Palo Alto, CA, USA). These metrics include maximum dose delivered to the AAA in Gray (Gy), minimum dose delivered in Gy, and mean dose delivered in Gy. The fractionation scheme and total dose applied in Gy were also recorded. Furthermore, all aneurysms were grouped according to the binary classification: infield vs. outfield. Infield aneurysms were located in the vicinity of the target of RT, i.e., the tumor, and subjected to varying degrees of radiation, while outfield aneurysms were located farther away and received no considerable (< 0.1 Gy) radiation exposure.

### Nonmalignancy AAA reference cohort

To obtain a reference cohort for statistical comparison, AAA growth data from two independent noncancer cohorts (Vienna and Stockholm AAA monitoring cohorts) were pooled, as reported before [9, 16, 17]. Briefly, all patients with ≥ 2 CT-based aneurysm diameter measurements over ≥ 6 months, no diagnosis of and treatment for cancer < 1 year before, during and < 1 year after the AAA growth data time-period, and an initial aortic diameter between 30–49 mm were included. Patients receiving steroid therapy for indications other than cancer were excluded. The observation time span was limited to 5 years. Thus, for all patients with follow-ups longer than 5 years, the aneurysm size at 5 years was calculated by linear interpolation in-between the closest measurements before and after the 5‑year boundary. This estimated 5‑year aneurysm diameter was then used in the last–first approximated growth rate calculations. No further adjustments were made.

### Statistical analysis

Where applicable values are shown as median (interquartile range: IQR) or average ± standard deviation (SD). All statistical analyses were performed using R version 4.0.3 (R Foundation, Vienna, Austria) and graphics were created using the *ggplot2* package. The level of significance was set at *p* < 0.05. For the comparison of two groups, a Welch’s t‑test was used, under the assumption of normal distribution. To assure comparability, only patients with an initial AAA diameter of 30–49 mm were used in all statistical analyses that compared different patient groups (RT vs. no-RT control vs. noncancer reference).

To account for the confounding effect of initial AAA diameter on AAA growth, a linear regression model was used to compare the growth rates among the different patient cohorts: rate = a × d_max0_ + est. × radiation + intercept.

Here, “est.” represents the estimated influence of the binary variable radiation (YES or NO) on AAA growth. It describes the increase or decrease of the annual AAA growth rate (mm/year) due to radiation. A similar model was used, to determine whether the amount of Gray that an AAA was subjected to affects its growth rate.

## Results

Over a 17-year period, 38 patients with AAA and concomitant malignancy (84% male, age 71 ± 7.3 years) who received well-documented RT met the inclusion criteria. Patient characteristics, comorbidities and medication are shown in Table 1. No aneurysm other than abdominal (36 infra-/2 juxtarenal) was detected.

The 38 patients had 47 malignancies (Suppl. Table 2). Prostate (34%), lung (18%), and head/neck cancer (16%) were the most frequent tumor entities. The majority of patients (66%) also received chemotherapy at some point (Suppl. Table 3). Overall, the average annual aneurysm growth in this AAA + cancer + RT cohort was 1.1 ± 1.3 mm (Table 2).

### AAA growth with respect to infield/outfield localization

**Fig. 1:**
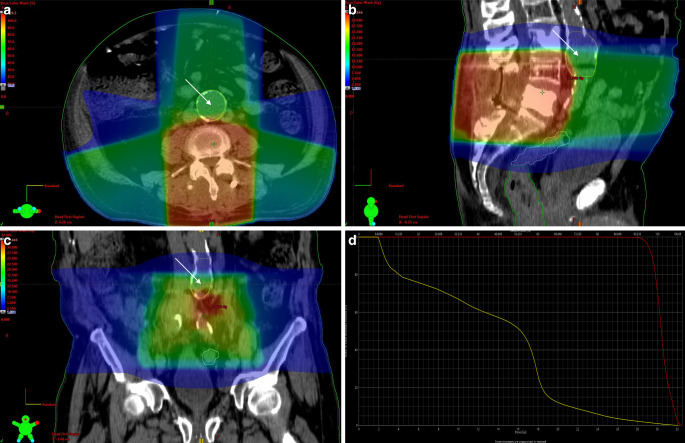
Dose color wash of radiation with contouring and relative radiation exposure of an infield abdominal aortic aneurysm (AAA). The radiation field was vertebrae L4–S1 for bone metastases in a patient with advanced prostate cancer. Target dose was 30 Gy (10 × 3 Gy). *Arrows* denote AAA location, highlighted (i.e., contoured) with a *yellow* frame. Mean AAA radiation exposure was calculated to be 13.4 Gy. **a** transversal view; **b** sagittal view; **c** frontal view; **d** relative radiation exposure of tumor and AAA. y‑axis: ratio of total structure volume (%), x‑axis: radiation dose (Gy); *curves* represent the target volume, i.e., the tumor (*red*) and the AAA (*yellow*) and demonstrate the uneven distribution of radiation exposure throughout different parts of the AAA

All AAAs included in the study have been manually contoured on the CT scans that were used for RT planning (Fig. 1, Suppl. Fig. 2). Eighteen AAAs that were located in proximity to the target of radiation were classified as “infield”, while 20 AAAs outside of the area that received considerable radiation were classified as “outfield”. The average annual growth rate of infield AAAs was lower compared to outfield AAAs, albeit not significantly (0.8 ± 1.0 vs. 1.3 ± 1.6 mm/year, *p* = 0.28; Table 2).

### AAA growth in RT and no-RT cancer patients

**Fig. 2:**
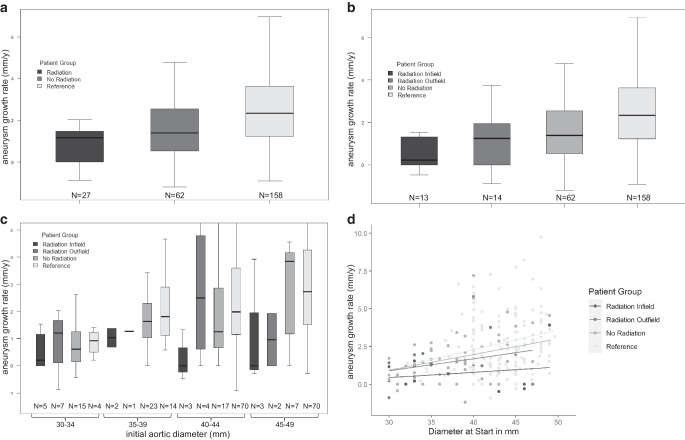
Comparison of annual abdominal aortic aneurysm (AAA) growth in radiation therapy (RT; infield/outfield), no-RT and noncancer reference cohorts. Boxplots demonstrate the median annual (y) AAA growth rate and interquartile ranges for patients with initial AAA diameter of 30–49 mm. Exact values for average and median annual growth rates for all cohorts are given in Table 2. (*N* =number of patients per group); **a** stratified by group; the difference between RT and reference was highly significant (*p* < 0.001); **b** stratified by group including infield/outfield; **c** stratified by group and initial AAA diameter; **d** linear regression analysis correcting for initial AAA diameter on the influence of RT on annual AAA growth rates. AAA growth was significantly reduced due to radiation in RT patients compared to the reference (est. = −1.02 mm/year, *p* = 0.03)

In the RT cohort, 27 of 38 patients had an initial AAA diameter of 30–49 mm and were eligible for further comparison to other groups. To this end, 62 AAA patients with an initial diameter of 30–49 mm and concomitant malignancy that had not received RT, but chemotherapy or surgery were identified using otherwise identical inclusion criteria (no-RT control; Table 1, Suppl. Table 2–3). In both groups, neither aneurysm- or cancer-surgery-related death nor AAA rupture occurred during the observation period. AAA growth was considerably reduced in the RT cohort compared to the no-RT control (Fig. 2a; 1.1 ± 1.5 vs. 1.8 ± 2.2 mm/year, *p* = 0.06). This growth reduction was more pronounced in infield (*n* = 13) as opposed to outfield (*n* = 14) aneurysms (Fig. 2b, Table 2).

### AAA growth in cancer and noncancer patients

AAA + cancer patients were compared to a noncancer AAA all-comers cohort with initial diameters 30–49 mm and an available follow-up time of up to 5 years (noncancer reference). In the reference cohort a higher proportion of female patients and smokers was seen (Table 1). Patients in the RT cohort showed a significantly reduced AAA growth rate compared to the noncancer reference (1.1 ± 1.5 vs. 2.7 ± 2.1 mm/year, *p* < 0.001; Fig. 2a, Table 2).

### AAA growth stratified by initial aneurysm diameter

When stratified for initial aneurysm diameter, infield aneurysms displayed the lowest annual growth rate in all AAA size classes (Fig. 2c). The AAA subclass with initial aneurysm diameter of 45–49 mm displayed significantly reduced AAA growth in RT vs. noncancer patients (1.1 ± 1.8 vs. 3.2 ± 2.4 mm/year, *p* = 0.04). Linear regression analyses correcting for initial AAA diameter reveal substantially reduced growth due to radiation for AAAs in RT patients compared to the no-RT control (est. = −0.63 mm/year, *p* = 0.17) and the noncancer reference (est. = −1.02 mm/year, *p* = 0.03; Fig. 2d). Furthermore, within the RT cohort, infield AAAs grew considerably slower than outfield AAAs (est. = −0.46 mm/year, *p* = 0.30; Fig. 2d).

### AAA growth with respect to radiation dosage

Different metrics for radiation exposure were calculated for all AAAs in the entire RT patient group (*n* = 38). Linear regression analyses correcting for initial AAA diameter showed no significant correlation between the amount of radiation exposure (both meanGy and maxGy) and AAA growth, with effect sizes being very small (meanGy: est. = −0.02 mm/year, *p* = 0.34; maxGy: est. = −0.01 mm/year, *p* = 0.41; Suppl. Fig. 3).

## Discussion

In this pilot exploration of RT on AAA + cancer patients, we found that RT might be associated with reduced AAA growth rates. This effect was observed in both infield and outfield patients, but more pronounced for patients whose AAA received direct radiation, although no general radiation dose-dependency was apparent (Fig. 2, Suppl. Fig. 3).

The association between RT and AAA growth reduction is an unexpected finding. In the literature, so far, RT has been primarily linked to an increased risk for cardiovascular disease [18–20]. RT for head and neck cancer is associated with the risk of carotid stenosis [21, 22]. Several studies report the formation of intracranial aneurysms as a consequence of RT [23–27]. On the other hand, RT has been reported to halt neoplastic aneurysm growth in rare cases (e.g., myxomatous aneurysms in the brain) [28].

The immune system plays a complex role in AAA formation and progression [29]. Traditionally, RT has been primarily associated with immunosuppressive effects [30]. According to this rationale, local radiation can cause systemic immunosuppression leading to a potential reduction of immune infiltration and, thus, an alleviation of inflammatory processes within the aneurysm wall that, in turn, might lead to a stabilizing effect on AAA growth in RT patients. An increasing number of studies regarding cancer therapy regimens that successfully combine RT and immunotherapy, however, argue for a more nuanced view [31–33]. Importantly, these studies also demonstrate abscopal (i.e., systemic) effects of RT that stimulate the immune response [34]. Accordingly, RT has the potential to influence immune infiltration and inflammatory processes in infield as well as outfield aneurysms in a complex fashion.

Since we can only speculate about the precise mode of action by which RT might lead to AAA stabilization, it would be very interesting to investigate the molecular and physiological effects of radiation on AAA growth in readily available murine models of AAA [35].

Naturally, reasons other than RT might influence AAA growth in cancer patients. In a recent publication we demonstrated a significant association between antimetabolite therapy and increased AAA growth, while administration of a variety of other chemotherapeutic agents showed trends for a possible AAA growth reduction [9]. Likewise, changes in lifestyle upon cancer diagnosis or cancer-related weight loss resulting in reduced metabolic syndrome might alter AAA growth. Inherently, indication for RT and AAA proximity to the target of radiation largely depend on cancer entity.

The study is limited by the small group size, especially with respect to initial aneurysm size, impeding statistical analysis and leading to possible type I errors with subgroups of only very few patients. Nevertheless, the use of a model accounting for initial aortic diameter is important, since growth and eventual rupture rates are diameter-dependent (Fig. 2c, [6, 14]). The retrospective identification of the study cohort constitutes an inherent bias. Aneurysms were identified based on CT report search, possibly lacking sufficient sensitivity upon diagnosis, whereas aneurysms in the reference cohort were pooled from aneurysm monitoring programs.

Since well-documented RT was relatively scarce in our institutional database due to many patients receiving RT in an external facility, the no-RT control cohort is larger in size. In addition, in being limited to a single-center analysis we were unable to generate datasets of AAA + cancer patients that match the size of the external noncancer reference cohort. Due to a low number of female patients, the results presented here cannot be generalized to the female population (Table 1).

Also, the cohorts presented here are relatively small and quite heterogenous with respect to the nature of their malignant disease and therapy combinations (Suppl. Table 2–3). Notably, all these features might interact in a complex fashion and obscure the statistical analysis of individual factors. Hence, further prospective research and multicenter approaches that allow for larger patient cohorts are needed to corroborate and refine the results presented here. If feasible, future studies should take additional predictors of AAA growth, such as AAA volume and AAA wall volume, into account [36]. Particularly for women, an indexing of aneurysm size measurements to body surface area could prove useful [37].

Especially in elderly multimorbid aneurysm patients, where surgery is associated with a high mortality risk, RT might present a much-needed noninvasive treatment alternative.

## Conclusion

The results from this study suggest that radiation therapy (RT) for cancer might be associated with reduced abdominal aortic aneurysm (AAA) growth. While this effect might be more pronounced in infield as opposed to outfield AAAs, the present data do not support a general radiation dose dependency. The potential stabilizing effect of RT on aneurysm progression could be a worthwhile investigation on a molecular level.

### Supplementary Information

Supplementary Figures 1–3

Supplementary Tables 1–4
