# Supplementary material for: Radiation therapy for cancer is potentially associated with reduced growth of concomitant abdominal aortic aneurysm
Source: Strahlenther Onkol. 2023 Sep 7;200(5):425–33. doi: 10.1007/s00066-023-02135-0 (PMC11039527; doi:10.1007/s00066-023-02135-0)
Supplement: Supplementary file 1 — Supplementary Figures 1–3 [file 66_2023_2135_MOESM1_ESM.pptx]

## Slide 1
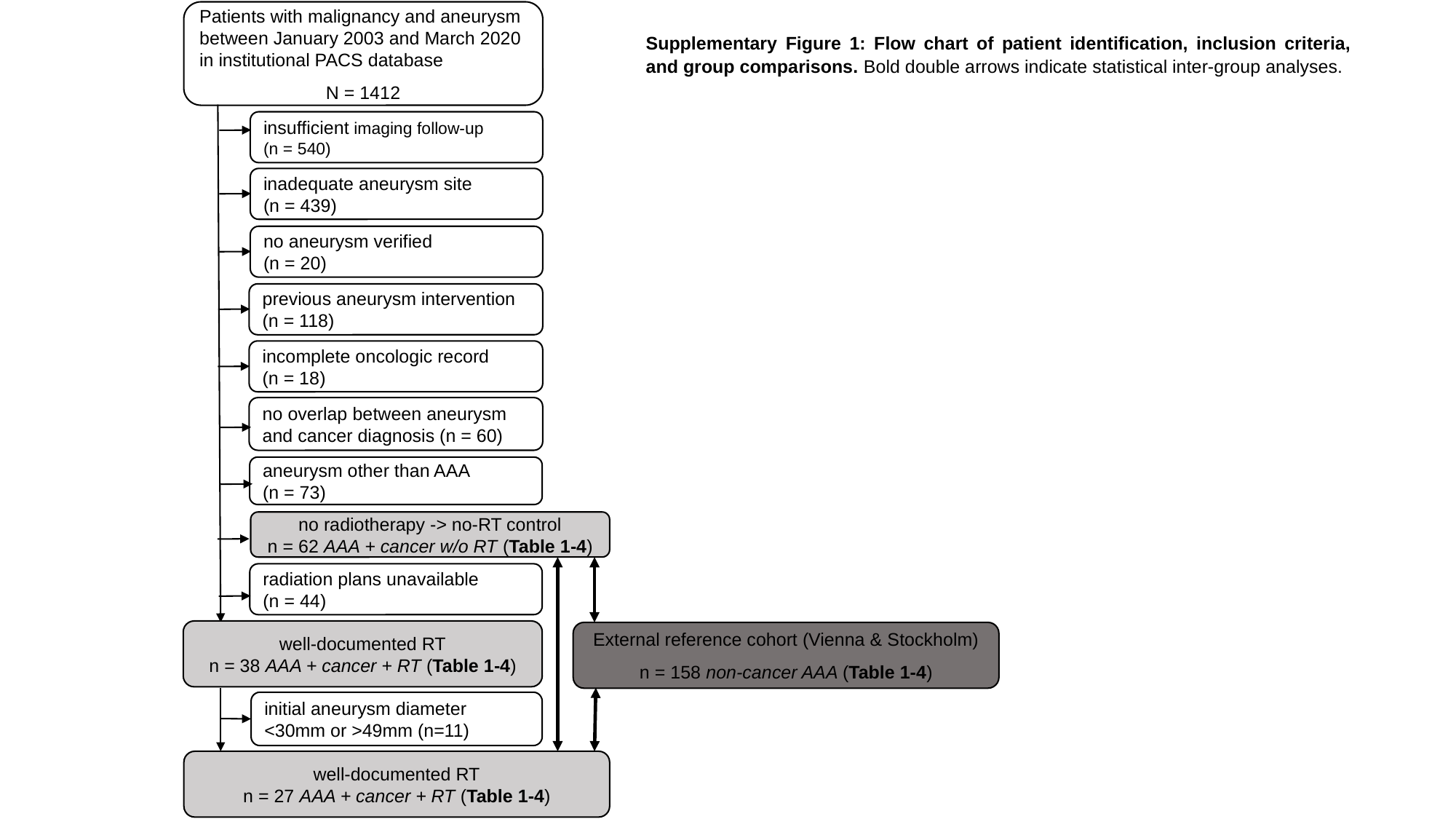

Patients with malignancy and aneurysm between January 2003 and March 2020 in institutional PACS database
N = 1412
insufficient imaging follow-up
(n = 540)
inadequate aneurysm site
(n = 439)
no aneurysm verified
(n = 20)
previous aneurysm intervention
(n = 118)
incomplete oncologic record
(n = 18)
no overlap between aneurysm and cancer diagnosis (n = 60)
aneurysm other than AAA
(n = 73)
no radiotherapy -> no-RT control
n = 62 AAA + cancer w/o RT (Table 1-4)
radiation plans unavailable
(n = 44)
well-documented RT
n = 38 AAA + cancer + RT (Table 1-4)
External reference cohort (Vienna & Stockholm)
n = 158 non-cancer AAA (Table 1-4)
initial aneurysm diameter <30mm or >49mm (n=11)
well-documented RT
n = 27 AAA + cancer + RT (Table 1-4)
Supplementary Figure 1: Flow chart of patient identification, inclusion criteria, and group comparisons. Bold double arrows indicate statistical inter-group analyses.

## Slide 2
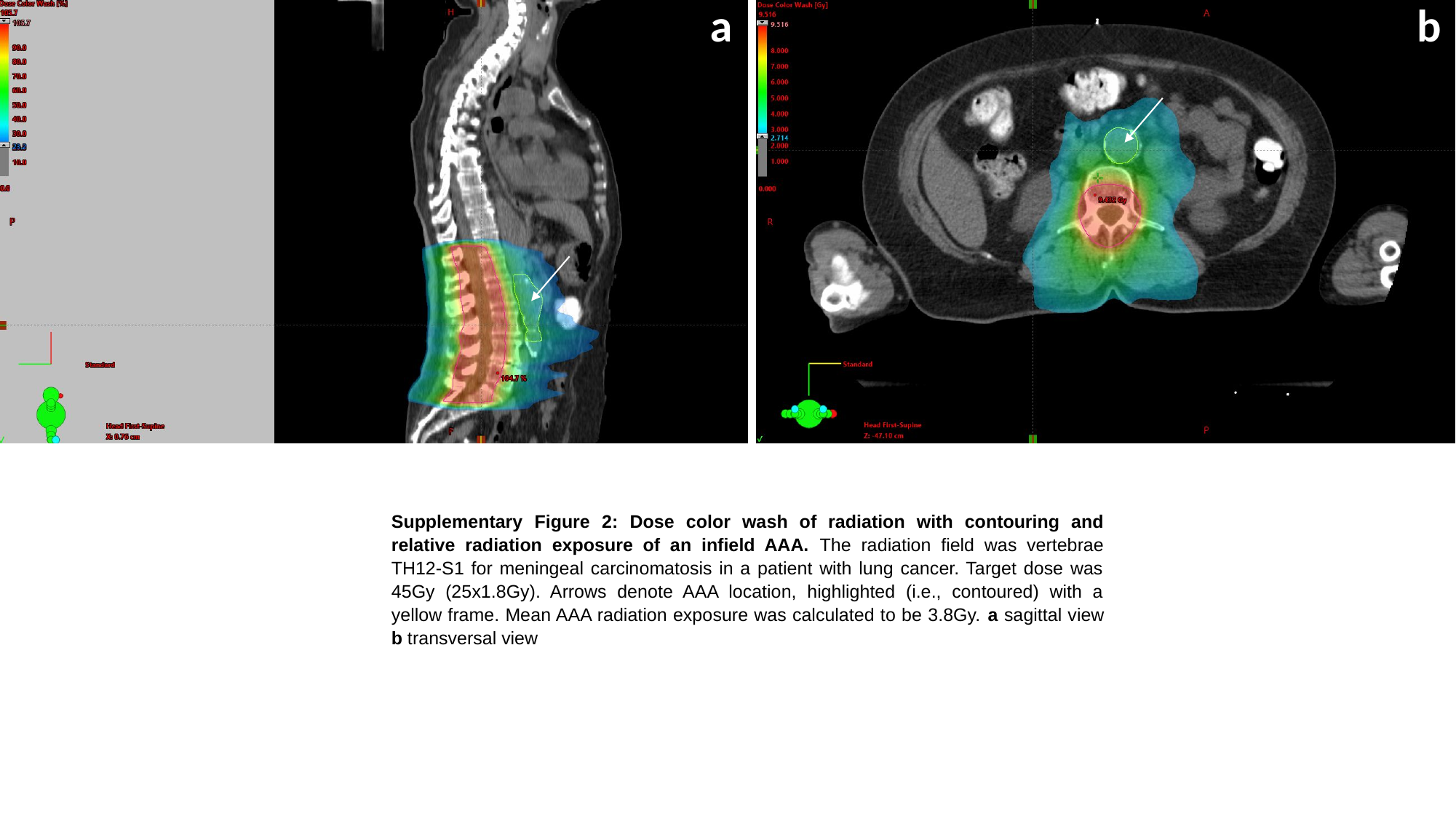

a
b
Supplementary Figure 2: Dose color wash of radiation with contouring and relative radiation exposure of an infield AAA. The radiation field was vertebrae TH12-S1 for meningeal carcinomatosis in a patient with lung cancer. Target dose was 45Gy (25x1.8Gy). Arrows denote AAA location, highlighted (i.e., contoured) with a yellow frame. Mean AAA radiation exposure was calculated to be 3.8Gy. a sagittal view b transversal view

## Slide 3
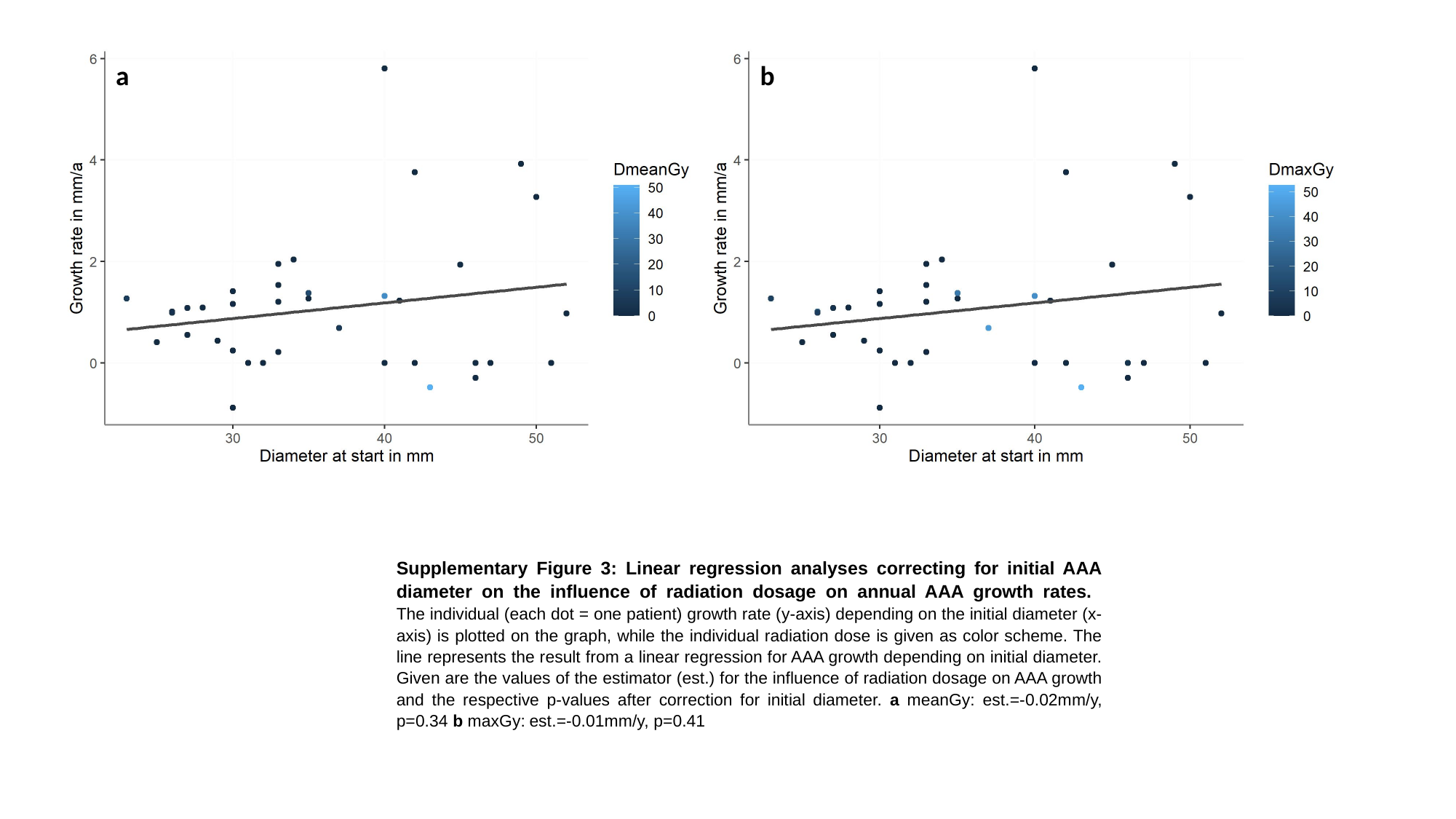

a
b
Supplementary Figure 3: Linear regression analyses correcting for initial AAA diameter on the influence of radiation dosage on annual AAA growth rates.
The individual (each dot = one patient) growth rate (y-axis) depending on the initial diameter (x-axis) is plotted on the graph, while the individual radiation dose is given as color scheme. The line represents the result from a linear regression for AAA growth depending on initial diameter. Given are the values of the estimator (est.) for the influence of radiation dosage on AAA growth and the respective p-values after correction for initial diameter. a meanGy: est.=-0.02mm/y, p=0.34 b maxGy: est.=-0.01mm/y, p=0.41
