# Supplementary material for: Radiation therapy for cancer is potentially associated with reduced growth of concomitant abdominal aortic aneurysm
Source: Strahlenther Onkol. 2023 Sep 7;200(5):425–33. doi: 10.1007/s00066-023-02135-0 (PMC11039527; doi:10.1007/s00066-023-02135-0)
Supplement: Supplementary file 2 — Supplementary Tables 1–4 [file 66_2023_2135_MOESM2_ESM.docx]

| **cancer summary** | **specific cancer types** |
| --- | --- |
| Colorectal | colon carcinoma, neuroendocrine tumor |
| Esophageal | esophageal carcinoma, adenocarcinoma of the esophago-gastric junction |
| Hematologic | diffuse large B-cell lymphoma (DLBCL), follicular lymphoma, Burkitt-lymphoma, anaplastic large cell lymphoma (ALCL), acute lymphoblastic leukemia (ALL), acute myeloid leukemia (AML), multiple myeloma (MM) |
| Prostate | prostate cancer |
| Kidney | renal cell carcinoma |
| Head and neck | glioblastoma IV, thyroid cancer |
| Lung | adenocarcinoma and squamous cell carcinoma from non-small cell lung cancer (NSCLC) |
| Skin | melanoma |
| Other | breast cancer, cancer of unknown primary origin, penis cancer, vulva cancer, Merkel-cell carcinoma, sarcoma |

**Supplementary Table 1: Summary and specific types of cancer.**

|  | **RT*** | | **infield*** | | **outfield*** | | **RT** | | **infield** | | **outfield** | | **no-RT** | |
| --- | --- | --- | --- | --- | --- | --- | --- | --- | --- | --- | --- | --- | --- | --- |
| **type of malignancy** | **n=38** | **%** | **n=18** | **%** | **n=20** | **%** | **n=27** | **%** | **n=13** | **%** | **n=14** | **%** | **n=62** | **%** |
|  |  |  |  |  |  |  |  |  |  |  |  |  |  |  |
| prostate | 13 | 34.2 | 9 | 50.0 | 4 | 20.0 | 9 | 33.3 | 7 | 53.8 | 2 | 14.3 | 11 | 17.7 |
| lung | 7 | 18.4 | 3 | 16.7 | 4 | 20.0 | 6 | 22.2 | 3 | 23.1 | 3 | 21.4 | 14 | 22.6 |
| esophagus | 4 | 10.5 | 1 | 5.6 | 3 | 15.0 | 4 | 14.8 | 1 | 7.7 | 3 | 21.4 | 9 | 14.5 |
| colorectal | 2 | 5.3 | 1 | 5.6 | 1 | 5.0 | 2 | 7.4 | 1 | 7.7 | 1 | 7.1 | 11 | 17.7 |
| hematologic | 4 | 10.5 | 2 | 11.1 | 2 | 10.0 | 1 | 3.7 | 0 | 0 | 1 | 7.1 | 6 | 9.7 |
| skin | 3 | 7.9 | 1 | 5.6 | 2 | 10.0 | 3 | 11.1 | 1 | 7.7 | 2 | 14.3 | 4 | 6.5 |
| head/neck | 6 | 15.8 | 2 | 11.1 | 4 | 20.0 | 5 | 18.5 | 2 | 15.4 | 3 | 21.4 | 0 | 0 |
| urinary bladder | 1 | 2.6 | 1 | 5.6 | 0 | 0 | 1 | 3.7 | 1 | 7.7 | 0 | 0 | 4 | 6.5 |
| kidney | 2 | 5.3 | 1 | 5.6 | 1 | 5.0 | 1 | 3.7 | 0 | 0 | 1 | 7.1 | 1 | 1.6 |
| bile duct | 0 | 0 | 0 | 0 | 0 | 0 | 0 | 0 | 0 | 0 | 0 | 0 | 3 | 4.8 |
| pancreas | 0 | 0 | 0 | 0 | 0 | 0 | 0 | 0 | 0 | 0 | 0 | 0 | 2 | 3.2 |
| other | 5 | 13.2 | 2 | 11.1 | 3 | 15.0 | 4 | 14.8 | 2 | 15.4 | 2 | 14.3 | 1 | 1.6 |
| >1 malignancy | 6 | 15.8 | 3 | 16.7 | 3 | 15.0 | 6 | 22.2 | 3 | 23.1 | 3 | 21.4 | 4 | 6.5 |

**Supplementary Table 2: Frequency of malignancies.** Percentages are given with respect to the total number of patients in the respective cohort. Cancer entities with only one instance overall have been summarized as “other”. Details on cancer classification are given in **Suppl. Table 1**. The RT cohorts marked with an asterisk include patients with an initial AAA diameter between 20-29mm and >49mm that subsequently have been removed for the comparison with the no-RT control and the reference.

|  | **RT*** | | **infield*** | | **outfield*** | | **RT** | | **infield** | | **outfield** | | **no-RT** | |
| --- | --- | --- | --- | --- | --- | --- | --- | --- | --- | --- | --- | --- | --- | --- |
| **chemotherapy** | **n=38** | **%** | **n=18** | **%** | **n=20** | **%** | **n=27** | **%** | **n=13** | **%** | **n=14** | **%** | **n=62** | **%** |
|  |  |  |  |  |  |  |  |  |  |  |  |  |  |  |
| alkylating agents | 10 | 26.3 | 3 | 16.7 | 7 | 35.0 | 7 | 25.9 | 2 | 15.4 | 5 | 35.7 | 27 | 43.5 |
| mitotic inhibitors | 6 | 15.8 | 2 | 11.1 | 4 | 20.0 | 3 | 11.1 | 1 | 7.7 | 2 | 14.3 | 14 | 22.6 |
| antiandrogens | 8 | 21.1 | 6 | 33.3 | 2 | 10.0 | 5 | 18.5 | 4 | 30.8 | 1 | 7.1 | 7 | 11.3 |
| antimetabolites | 10 | 26.3 | 5 | 27.8 | 5 | 25.0 | 8 | 29.6 | 4 | 30.8 | 4 | 28.6 | 20 | 32.3 |
| GnRH agonists | 7 | 18.4 | 6 | 33.3 | 1 | 5.0 | 5 | 18.5 | 4 | 30.8 | 1 | 7.1 | 5 | 8.1 |
| corticosteroids | 4 | 10.5 | 2 | 11.1 | 2 | 10.0 | 2 | 7.4 | 0 | 0 | 2 | 14.3 | 3 | 4.8 |
| monoclonal antibodies | 4 | 10.5 | 2 | 11.1 | 2 | 10.0 | 3 | 11.1 | 2 | 15.4 | 1 | 7.1 | 7 | 11.3 |
| anthracycline | 2 | 5.3 | 1 | 5.6 | 1 | 5.0 | 2 | 7.4 | 1 | 7.7 | 1 | 7.1 | 5 | 8.1 |
| PSMA ligands | 2 | 5.3 | 1 | 5.6 | 1 | 5.0 | 2 | 7.4 | 1 | 7.7 | 1 | 7.1 | 1 | 1.6 |
| kinase/proteasome inhibitors | 4 | 10.5 | 2 | 11.1 | 2 | 10.0 | 2 | 7.4 | 0 | 0 | 2 | 14.3 | 4 | 6.5 |
| topoisomerase inhibitors | 3 | 7.9 | 2 | 11.1 | 1 | 5.0 | 3 | 11.1 | 2 | 15.4 | 1 | 7.1 | 8 | 12.9 |
| immunecheckpoint inhibitors | 2 | 5.3 | 1 | 5.6 | 1 | 5.0 | 1 | 3.7 | 0 | 0 | 1 | 7.1 | 2 | 3.2 |
| IMiDs | 3 | 7.9 | 2 | 11.1 | 1 | 5.0 | 1 | 3.7 | 0 | 0 | 1 | 7.1 | 2 | 3.2 |
| antifolates | 1 | 2.6 | 0 | 0 | 1 | 5.0 | 0 | 0 | 0 | 0 | 0 | 0 | 1 | 1.6 |
| VEGF inhibitors | 1 | 2.6 | 1 | 5.6 | 0 | 0 | 1 | 3.7 | 1 | 7.7 | 0 | 0 | 4 | 6.5 |
| aromatase inhibitors | 0 | 0.0 | 0 | 0 | 0 | 0 | 0 | 0 | 0 | 0 | 0 | 0 | 1 | 1.6 |
| HIFU | 1 | 2.6 | 1 | 5.6 | 0 | 0 | 0 | 0 | 0 | 0 | 0 | 0 | 0 | 0 |
| no chemo | 13 | 34.2 | 6 | 33.3 | 7 | 35.0 | 11 | 40.7 | 6 | 46.2 | 5 | 35.7 | 17 | 27.4 |

**Supplementary Table 3: Frequency of chemotherapies.** Percentages are given with respect to the total number of patients in the respective cohort. Most patients received multiple classes of chemotherapeutic agents. The RT cohorts marked with an asterisk include patients with an initial AAA diameter between 20-29mm and >49mm that subsequently have been removed for the comparison with the no-RT control and the reference.

|  |  | **RT (n=38) *** | | | |
| --- | --- | --- | --- | --- | --- |
|  |  | growth rate (mm/y) | | follow-up time (y) | |
|  | # | mean±sd | median (Q1-Q3) | mean±sd | median (Q1-Q3) |
| 20-29mm | 8 | 0.85±0.34 | 1.00 (0.52 – 1.09) | 8.90±2.54 | 8.87 (7.83 – 9.97) |
| 30-34mm | 12 | 0.74±0.93 | 0.70 (0.00 – 1.44) | 4.06±3.94 | 2.09 (1.26 – 5.63) |
| 35-39mm | 3 | 1.11±0.37 | 1.27 (0.98 – 1.32) | 5.76±1.36 | 5.82 (5.09 – 6.45) |
| 40-44mm | 7 | 1.66±2.31 | 1.22 (0.00 – 2.54) | 4.14±2.34 | 2.91 (2.43 – 5.42) |
| 45-49mm | 5 | 1.11±1.80 | 0.00 (0.00 – 1.93) | 2.77±0.88 | 3.26 (2.59 – 3.12) |
| >=50mm | 3 | 1.41±1.68 | 0.97 (0.49 – 2.12) | 1.16±0.62 | 1.03 (0.82 – 1.43) |
| **total** | **38** | **1.06±1.33** | **1.00 (0.00 – 1.36)** | **4.83±3.54** | **3.78 (2.06 – 7.59)** |

|  |  | **infield (n=18) *** | | | |
| --- | --- | --- | --- | --- | --- |
|  |  | growth rate (mm/y) | | follow-up time (y) | |
|  | # | mean±sd | median (Q1-Q3) | mean±sd | median (Q1-Q3) |
| 20-29mm | 5 | 0.96±0.31 | 1.01 (0.99 – 1.08) | 8.66±2.57 | 8.66 (8.30 – 9.24) |
| 30-34mm | 5 | 0.58±0.72 | 0.21 (0.00 – 1.16) | 4.80±3.84 | 4.93 (1.30 – 7.75) |
| 35-39mm | 2 | 1.03±0.48 | 1.03 (0.86 – 1.20) | 5.09±1.02 | 5.09 (4.73 – 5.45) |
| 40-44mm | 3 | 0.28±0.93 | 0.00 (-0.24 – 0.66) | 3.68±2.10 | 2.91 (2.49 – 4.48) |
| 45-49mm | 3 | 1.21±2.35 | 0.00 (-0.15 – 1.96) | 3.31±0.05 | 3.32 (3.29 – 3.34) |
| >=50mm |  |  |  |  |  |
| **total** | **18** | **0.79±1.01** | **0.84 (0.00 – 1.24)** | **5.47±3.19** | **4.94 (3.27 – 8.16)** |

|  |  | **outfield (n=20) *** | | | |
| --- | --- | --- | --- | --- | --- |
|  |  | growth rate (mm/y) | | follow-up time (y) | |
|  | # | mean±sd | median (Q1-Q3) | mean±sd | median (Q1-Q3) |
| 20-29mm | 3 | 0.68±0.36 | 0.55 (0.48 – 0.82) | 9.30±2.99 | 9.07 (7.75- 10.73) |
| 30-34mm | 7 | 0.85±1.09 | 1.20 (0.12 – 1.68) | 3.53±4.22 | 2.05 (1.39 – 3.16) |
| 35-39mm | 1 | 1.27±/ | 1.27 (1.27 – 1.27) | 7.09±/ | 7.09 (7.09 – 7.09) |
| 40-44mm | 4 | 2.70±2.60 | 2.49 (0.92 – 4.27) | 4.48±2.76 | 3.62 (2.44 – 5.66) |
| 45-49mm | 2 | 0.97±1.37 | 0.97 (0.48 – 1.45) | 1.94±0.91 | 1.94 (1.62 – 2.26) |
| >=50mm | 3 | 1.41±1.68 | 0.97 (0.49 – 2.12) | 1.16±0.62 | 1.03 (0.82 – 1.43) |
| **total** | **20** | **1.31±1.55** | **1.14 (0.18 – 1.94)** | **4.25±3.82** | **2.43 (1.57 – 6.60)** |

|  |  | **RT (n=27)** | | | |
| --- | --- | --- | --- | --- | --- |
|  |  | growth rate (mm/y) | | follow-up time (y) | |
|  | # | mean±sd | median (Q1-Q3) | mean±sd | median (Q1-Q3) |
| 20-29mm |  |  |  |  |  |
| 30-34mm | 12 | 0.74±0.93 | 0.70 (0.00 – 1.44) | 4.06±3.94 | 2.09 (1.26 – 5.63) |
| 35-39mm | 3 | 1.11±0.37 | 1.27 (0.98 – 1.32) | 5.76±1.36 | 5.82 (5.09 – 6.45) |
| 40-44mm | 7 | 1.66±2.31 | 1.22 (0.00 – 2.54) | 4.14±2.34 | 2.91 (2.43 – 5.42) |
| 45-49mm | 5 | 1.11±1.80 | 0.00 (0.00 – 1.93) | 2.77±0.88 | 3.26 (2.59 – 3.12) |
| >=50mm |  |  |  |  |  |
| **total** | **27** | **1.09±1.50** | **1.16 (0.00 – 1.47)** | **4.03±2.96** | **3.26 (2.06 – 5.37)** |

|  |  | **infield (n=13)** | | | |
| --- | --- | --- | --- | --- | --- |
|  |  | growth rate (mm/y) | | follow-up time (y) | |
|  | # | mean±sd | median (Q1-Q3) | mean±sd | median (Q1-Q3) |
| 20-29mm |  |  |  |  |  |
| 30-34mm | 5 | 0.58±0.72 | 0.21 (0.00 – 1.16) | 4.80±3.84 | 4.93 (1.30 – 7.75) |
| 35-39mm | 2 | 1.03±0.48 | 1.03 (0.86 – 1.20) | 5.09±1.02 | 5.09 (4.73 – 5.45) |
| 40-44mm | 3 | 0.28±0.93 | 0.00 (-0.24 – 0.66) | 3.68±2.10 | 2.91 (2.49 – 4.48) |
| 45-49mm | 3 | 1.21±2.35 | 0.00 (-0.15 – 1.96) | 3.31±0.05 | 3.32 (3.29 – 3.34) |
| >=50mm |  |  |  |  |  |
| **total** | **13** | **0.73±1.18** | **0.21 (0.00 – 1.32)** | **4.24±2.51** | **3.37 (2.91 – 5.82)** |

|  |  | **outfield (n=14)** | | | |
| --- | --- | --- | --- | --- | --- |
|  |  | growth rate (mm/y) | | follow-up time (y) | |
|  | # | mean±sd | median (Q1-Q3) | mean±sd | median (Q1-Q3) |
| 20-29mm |  |  |  |  |  |
| 30-34mm | 7 | 0.85±1.09 | 1.20 (0.12 – 1.68) | 3.53±4.22 | 2.05 (1.39 – 3.16) |
| 35-39mm | 1 | 1.27±/ | 1.27 (1.27 – 1.27) | 7.09±/ | 7.09 (7.09 – 7.09) |
| 40-44mm | 4 | 2.70±2.60 | 2.49 (0.92 – 4.27) | 4.48±2.76 | 3.62 (2.44 – 5.66) |
| 45-49mm | 2 | 0.97±1.37 | 0.97 (0.48 – 1.45) | 1.94±0.91 | 1.94 (1.62 – 2.26) |
| >=50mm |  |  |  |  |  |
| **total** | **14** | **1.42±1.72** | **1.25 (0.06 – 1.94)** | **3.83±3.41** | **2.43 (1.76 – 4.64)** |

|  |  | **no-RT control (n=62)** | | | |
| --- | --- | --- | --- | --- | --- |
|  |  | growth rate (mm/y) | | follow-up time (y) | |
|  | # | mean±sd | median (Q1-Q3) | mean±sd | median (Q1-Q3) |
| 20-29mm |  |  |  |  |  |
| 30-34mm | 15 | 0.74±0.80 | 0.61 (0.15 – 1.26) | 4.04±3.70 | 2.39 (1.61 – 4.78) |
| 35-39mm | 23 | 2.00±2.73 | 1.63 (1.03 – 2.29) | 3.41±3.00 | 2.42 (1.08 – 4.77) |
| 40-44mm | 17 | 2.08±2.13 | 1.25 (0.67 – 2.86) | 3.25±3.07 | 2.09 (0.97 – 3.64) |
| 45-49mm | 7 | 2.72±1.88 | 3.85 (1.17 – 4.16) | 2.74±2.26 | 1.88 (1.35 – 4.06) |
| >=50mm |  |  |  |  |  |
| **total** | **62** | **1.80±2.19** | **1.39 (0.54 – 2.53)** | **4.45±3.09** | **2.32 (1.04 – 4.31)** |

|  |  | **non-cancer reference (n=158)** | | | |
| --- | --- | --- | --- | --- | --- |
|  |  | growth rate (mm/y) | | follow-up time (y) | |
|  | # | mean±sd | median (Q1-Q3) | mean±sd | median (Q1-Q3) |
| 20-29mm |  |  |  |  |  |
| 30-34mm | 3 | 0.86±0.50 | 0.92 (0.65 – 1.13) | 1.97±1.55 | 1.65 (0.86 – 2.76) |
| 35-39mm | 14 | 2.32±1.69 | 1.81 (1.13 – 2.80) | 2.57±1.28 | 2.89 (1.77 – 3.37) |
| 40-44mm | 70 | 2.45±1.73 | 1.98 (1.16 – 3.59) | 3.64±1.25 | 3.97 (2.97 – 4.82) |
| 45-49mm | 70 | 3.18±2.41 | 2.72 (1.56 – 4.24) | 2.96±1.21 | 2.97 (2.06 – 3.92) |
| >=50mm |  |  |  |  |  |
| **total** | **158** | **2.72±2.08** | **2.33 (1.25 – 3.64)** | **3.21±1.30** | **3.27 (2.36 – 4.15)** |

**Supplementary Table 4: Number of patients, annual aneurysm growth rates and follow-up time for all study cohorts with respect to initial aneurysm diameter size classes.** The RT cohorts marked with an asterisk include patients with an initial AAA diameter between 20-29mm and >49mm that subsequently have been removed for the comparison with the no-RT control and the reference.
